# Meta’omics: Challenges and Applications

**DOI:** 10.3390/ijms23126486

**Published:** 2022-06-10

**Authors:** Valerio Fulci

**Affiliations:** Dipartimento di Medicina Molecolare, Università di Roma “La Sapienza”, 00161 Rome, Italy; valerio.fulci@uniroma1.it

Metagenomics and metatranscriptomics are emerging as key disciplines towards a fully understanding the complex relationships between living organisms belonging to different kingdoms. These approaches, leveraging state-of-the-art NGS techniques, allow for an unbiased, high-throughput characterization of complex communities. Thus far, metagenomic approaches have been exploited in several fields including ecology, medicine and biotechnology. Multi-omic approaches combining the output of metagenomics analyses with further omic technologies proved to be informative, for example in the study of inflammatory bowel disease (IBD) [1].

However, many metagenomics datasets are possibly not yet fully explored due to limited analysis and/or a lack of comprehensive annotation information at the time of analysis. Notably, it has been demonstrated that most RNA-seq datasets contain a significant amount of reads that could be further investigated to characterize microbiomes and viromes using proper metatranscriptomics pipelines [2]. A computational analysis of metagenomics data obtained may also fail to capture relevant aspects due to an inappropriate choice of reference database as well as challenges in the de novo assembly of metagenomes and biases in annotation algorithms.

The increasing complexity of the metagenomic datasets produced as well as the escalating opportunities to integrate metagenomic datasets with other high-throughput databases require further development of computational methods to extract meaningful information from multi-omic databases. Innovative approaches include dedicated implementations of machine learning algorithms as well as the application of algorithms originally developed in other fields, such as network analysis. Furthermore, simpler interfaces are required to make complex computational tools available to a larger cohort of researchers, thus boosting research and increasing the standardization of protocols and opportunities for data comparison. 

The papers published in this Special Issue highlight the wide range of different disciplines, ranging from ecology to oncology, that can take advantage of metagenomics and metatranscriptomic approaches. Furthermore, the data presented witness the power of conceptually innovative analytical methods in the field of metagenomics.

Nardelli and co-workers [3] provided an example of the integration of 16S amplicon sequencing data with other biomarkers. They unveiled a correlation between circulating CCL2, microbiome composition and Colon–Rectal Cancer (CRC) disease status, leading to the hypothesis that an interplay between microbiota and inflammation may contribute to the onset of CRC.

A multi-omics approach was instead exploited by a different group [4] to integrate gut microbiome metagenomics, metabolomics and response to therapy in non-small cell lung cancer patients. Their innovative analysis pipeline, relying on a network-based approach, highlighted interesting associations deserving further investigation to validate novel biomarkers potentially useful in NSCLC patient management and stratification.

Scrhenzel and co-workers [5] highlighted that shotgun metagenomic outcompetes previous techniques for the detection of infectious agents in a clinical setting, thus allowing for the identification of Mycobacterium chelonae infection possibly related to a case of acute contained rupture of a biological composite graft. 

Jo and colleagues [6] instead exploited existing RNA-seq data to carry out an extensive characterization of the garlic microbiome. Their approach highlights that reanalysis of existing datasets on different premises is a valid approach to metagenomics. Furthermore, the authors compared the performance of state-of-the-art computational tools, highlighting the limitations of k-mer-based approaches in the analysis of plant viromes.

Finally, in this issue, Ansorge and colleagues presented a novel modular pipeline, namely dadaist2 [7], to analyze amplicon sequencing datasets. Dadaist2 provides a simple interface to the widely used DADA2 module for raw data denoising and integrates several other modules, including a dedicated tool developed by the authors to manage variable length amplicons, such as fungal Internal Transcribed Spacer (ITS) data, filling an existing gap in eukaryotic metagenomics data analysis.
